# A thermal after-effect of UV irradiation of muscle glycogen phosphorylase *b*

**DOI:** 10.1371/journal.pone.0189125

**Published:** 2017-12-07

**Authors:** Valeriya V. Mikhaylova, Tatiana B. Eronina, Natalia A. Chebotareva, Sergey Yu. Kleymenov, Vladimir V. Shubin, Boris I. Kurganov

**Affiliations:** 1 Department of Structural Biochemistry of Proteins, Bach Institute of Biochemistry, Federal State Institution “Federal Research Centre “Fundamentals of Biotechnology” of the Russian Academy of Sciences”, Moscow, Russia; 2 Kol’tsov Institute of Developmental Biology, Russian Academy of Sciences, Moscow, Russia; Kermanshah University of Medical Sciences, ISLAMIC REPUBLIC OF IRAN

## Abstract

Different test systems are used to characterize the anti-aggregation efficiency of molecular chaperone proteins and of low-molecular-weight chemical chaperones. Test systems based on aggregation of UV-irradiated protein are of special interest because they allow studying the protective action of different agents at physiological temperatures. The kinetics of UV-irradiated glycogen phosphorylase *b* (UV-Ph*b*) from rabbit skeletal muscle was studied at 37°C using dynamic light scattering in a wide range of protein concentrations. It has been shown that the order of aggregation with respect to the protein is equal to unity. A conclusion has been made that the rate-limiting stage of the overall process of aggregation is heat-induced structural reorganization of a UV-Ph*b* molecule, which contains concealed damage.

## Introduction

One of the important problems of modern biochemistry and molecular biology is shedding light on the mechanism of anti-aggregation activity of molecular chaperones (small heat-shock proteins) and low-molecular-weight osmolytes acting as chemical chaperons [1]. Test systems used for screening of agents possessing protective action are based on aggregation of client proteins induced by heating or by reduction of disulphide bonds in the protein molecule. The aggregation process in these test systems involves a stage of unfolding of the protein molecule and a stage of aggregation of the denatured protein. In general it is difficult to discriminate between the effects caused by the agents under study on the unfolding stage or the aggregation stage. To overcome this drawback, we have proposed to use aggregation of UV-irradiated proteins (glycogen phosphorylase *b* (Ph*b*) and glyceraldehyde-3-phosphate dehydrogenase (GAPHD) from rabbit skeletal muscles) as test systems for studying anti-aggregation activity of chaperones [1–3]. In such test systems we are studying aggregation of UV-denatured proteins, and it is expected that the direct action of chaperones on the stage of aggregation can be estimated. However, the kinetic mechanism of aggregation of UV-irradiated proteins remains unknown.

In the present work we have studied the kinetics of UV-irradiated Ph*b* (UV-Ph*b*) at 37°C using dynamic light scattering (DLS). To control the denaturation process, enzyme assay and differential scanning calorimetry (DSC) methods were used. To establish the kinetic mechanism of the aggregation process, aggregation kinetics was studied at various protein concentrations. It was remarkable to discover that the kinetics of UV-Ph*b* aggregation followed kinetics of the reaction of the first order. This is to say that the aggregation process involves a relatively slow stage of heat-induced structural reorganization of UV-Ph*b* molecule, which contains concealed damage, followed by fast aggregation of the transformed molecules. The thermal after-effect of UV irradiation of Ph*b* was confirmed by the data on circular dichroism (CD).

## Materials and methods

### Materials

Hepes, glucose 1-phosphate, AMP were purchased from “Sigma” (USA), NaCl was purchased from “Reakhim” (Russia), glycogen was purchased from “Olaine” (Latvia). All solutions for the experiments were prepared using deionized water obtained with the Easy-Pure II RF system (Barnstead, USA).

### Isolation of Ph*b* and enzyme assay

Ph*b* was purified from rabbit skeletal muscle as described earlier [4]. Preparations of the enzyme were electrophoretically homogeneous. Prior to the experiments, the enzyme was passed through the column of Sephadex G-15 equilibrated with 0.03 M Hepes buffer, pH 6.8, containing 0.1 M NaCl. All experiments were carried out in this buffer. Ph*b* concentration was determined spectrophotometrically at 280 nm using the absorbance coefficient $A_{\mathrm{cm}}^{1\%}$ of 13.2 [5]. The enzymatic activity of Ph*b* was determined by the turbidimetric method [6,7], which is based on registration of an increase in glycogen solution absorbance at 360 nm using 1 cm cuvettes and ThermoSpectronic model Genesys 6 spectrophotometer (USA) equipped with a thermostatically controlled cell. Spectrophotometric data were recorded using an IBM-compatible computer. The kinetics of the enzymatic reaction was registered at 30°C. The reaction mixture contained glycogen 0.25 mg/ml, 1 mM AMP and 30 mM glucose 1-phosphate. The reaction was initiated by addition of the enzyme to the reaction mixture.

### UV irradiation of Ph*b*

UV irradiation of Ph*b* (1.5 mg/ml) was carried out in 1 cm path quartz cell at 6°C. The equipment with Hg-Xe Lamp L8252 (Hamamatsu Photonics, Japan) was used in the irradiation experiments. The power of incident light was 10.4 mW/cm^2^. The samples of UV-Ph*b* were centrifuged at 12,850 × *g* for 10 min at 4°C.

### Thermal denaturation of UV-irradiated Ph*b*

Thermal denaturation of UV-Ph*b* solutions (1.5 mg/ml) was investigated by DSC using the adiabatic scanning microcalorimeter NanoDSC (TA Instruments New Castle, DE, USA) with 0.3 ml capillary platinum cells at the rate of heating equal to 1°C/min using the temperature range from 10 to 90°C and constant pressure of 3.0 atm. The dependence of the excess heat capacity on temperature was calculated using Origin software (MicroCal, Inc., USA). A portion of Ph*b* remaining in the native state after UV-irradiation was estimated as the ratio of the area under the DSC profile for UV-Ph*b* to that for intact Ph*b*.

### Aggregation kinetics studies

The kinetics of thermal aggregation of UV-Ph*b* at 37°C was studied by registration of an increment in the light scattering intensity using a commercial Photocor Complex (Photocor Instruments, Inc., USA) with a He-Ne laser (Coherent, USA, Model 31–2082, 632.8 nm, 10 mW) as a light source. DynaLS software (Alango, Israel) was employed for polydisperse analysis of DLS data. The diffusion coefficient *D* of the particles is directly related to the decay rate τ_c_ of the time-dependent correlation function for the light scattering intensity fluctuations: *D* = 1/2τ_c_*k*^2^. In this equation *k* is the wave number of the scattered light, *k* = (4π*n*/λ)sin(θ/2), where *n* is the refractive index of the solvent, λ is the wavelength of the incident light in vacuum and θ is the scattering angle. The mean hydrodynamic radius of the particles, *R*_h_, can then be calculated according to the Stokes–Einstein equation: *D* = *k*_B_*T*/6πη*R*_h_, where *k*_B_ is Boltzmann’s constant, *T* is the absolute temperature and η is the dynamic viscosity of the solvent [8]. The value of the refractive index of 0.03 M Hepes buffer, pH 6.8, containing 0.1 M NaCl was determined in ABBEMAT 500 refractometer (Anton Paar, Austria) at 37°C. Density of this buffer was determined in density meter DMA 4500 (Anton Paar, Austria) at 37°C. Dynamic viscosity of the buffer was determined in automatic microviscometer (Anton Paar, Austria) in system 1.6/1.500 mm at 37°C. The value of refractive index (1.3333 ± 0.0001) and dynamic viscosity of buffer solution (0.7271 ± 0.0005 mPa·s) were used for the determination of the values of the hydrodynamic radius (*R*_h_) of protein aggregates in the dynamic light scattering measurements. Calculations of the hydrodynamic radius of the protein aggregates followed the procedures described in the previous publications [2,4,9].

To characterize the accumulation of large particles in the course of protein aggregation, the experimentally measured intensity autocorrelation functions, which correspond to z-average particle sizes, were used. The aggregation process was initiated by addition of an aliquot of protein to the final volume of 0.5 ml.

### Analytical ultracentrifugation

Sedimentation velocity experiments were carried out at 25°C in a Model E analytical ultracentrifuge (Beckman), equipped with absorbance optics, a photoelectric scanner, a monochromator and a computer on-line. A four-hole rotor An-F Ti and 12 mm double sector cells were used. The rotor was placed into a thermostat at 25°C for the night before the run. Samples of UV-Ph*b* were heated at 37°C for 90 min and cooled to 25°C before the runs. The rotor speed was 15,000 rpm in the first run and 48,000 rpm in subsequent runs. Sedimentation profiles of the proteins were recorded by measuring the absorbance at 288 nm. All cells were scanned simultaneously. The time interval between scans was 2.5 min. The differential sedimentation coefficient distributions [*c*(*s*) versus *s*] were determined using SEDFIT program [10]. The *c*(*s*) analysis was performed with regularization at the confidence level of 0.68 and a floating frictional ratio. Weight-average sedimentation coefficients were obtained by integration of the *c*(*s*) distribution. Sedimentation coefficients were corrected to the standard conditions (a solvent with the density and viscosity of water at 20°C) using SEDFIT and SEDNTERP [11] programs.

### CD spectroscopy

CD spectra of Ph*b* (1 mg/ml) and UV-Ph*b* (1 mg/ml) in the region of 182–300 nm were recorded on the Chirascan spectropolarimeter equipped with a thermoelectric temperature control unit (Applied Photophysics Ltd., Surrey, UK) at 10°C in a 0.01 cm cell. The ellipticity was recorded every 1-nm and averaged over 3 s of acquisition time. Spectral bandwidth was equal to 1.5 nm. Protein concentration was determined by the absorbance value at 205 nm [12]. All CD measurements described here were made using Quartz Suprasil 0.01 cm cells (Helma Analytics). Protein secondary structure determinations were obtained using the DICHROWEB server [13] with the CDSSTR analysis program and assuming SP180 as the dataset reference [14].

Thermal unfolding experiments were carried out by measuring molar ellipticity of Ph*b* (0.17 mg/ml) and UV-Ph*b* (0.17 mg/ml) in 0.1 cm cell at 220 nm over the temperature range from 10 to 90°C at a constant heating rate of 1°C/min. All measurements were performed and repeated at least three times.

### Determination of the fraction of the aggregated protein

Samples of UV-Ph*b* (0.3 mg/ml) were incubated at 37°C in a solid state thermostat Bio-TBD-120 “Biosan” (Latvia). To determine the amount of the aggregated protein at 37°C, aliquots of 0.15 ml were taken from the same tube at appropriate time intervals, immediately placed in ice water bath and centrifuged for 15 min at 16,350 × *g*. The optical density (OD) of the supernatant was measured at 280 nm. The fraction of the aggregated protein at 37°C (γ_agg_) was calculated as (1 –OD/OD_0_), where OD_0_ was the optical density of the unheated solution. Samples of the unheated protein served as the control. The concentration of the aggregated protein [UV-Ph*b*_agg_] was calculated as a product of γ_agg_ and the initial protein concentration. The measurements were performed three times. The error in estimation of γ_agg_ was 2%.

### Data analysis

Origin Pro 8.0 SR0 software was used for the calculations. To characterize the degree of agreement between experimental data and calculated values, we used the coefficient of determination *R*^2^ (see [15]).

### Ethics statement

The animal experiments followed the legal and ethical guidelines as indicated in Directive 2010/63/EU of the European Parliament and the European Council of September 22, 2010 on protection of animals used for scientific purposes. The experimental protocol No. 11 of March 10, 2016 was approved by the Ethics Committees for Animal Research of the Kol’tzov Institute of Developmental Biology of Russian Academy of Sciences in accordance with the Regulations for Laboratory Practice in Russian Federation. All surgeries were performed under anesthesia (isoflurane gas) and all effots were made to avoid suffering. Euthanasia was carried out by inserting a 25 mm x 0·65 mm (23 gauge) needle through a lateral ear vein. Intravenous sodium pentobarbital (Nembutal: Abbott Laboratories, Madrid) at 30 mg kg-1 as a 2% solution.

## The theory

### Kinetics of protein aggregation

Protein aggregation can be considered as an irreversible reaction proceeding with participation of *n* molecules of a non-aggregated protein P [16]:

$$nP\overset{k}{\to }P_{\mathrm{agg}}$$ (1)

(P_agg_ is the aggregated form of the protein, *k* is the rate constant of the *n*-th order). When analyzing the kinetic curves of aggregation registered by measuring an increase in light scattering intensity (*I*) in time, it is assumed that the *I* value is proportional to the concentration of the aggregated protein: *I* = ε[P_agg_]. The expression for the rate of change in the light scattering intensity has the following form [16]:

$$\frac{d\left(I-I_{0}\right)}{dt}=\frac{k{\left[P\right]}_{0}^{n-1}}{{\left(I_{\lim}-I_{0}\right)}^{n-1}}{\left(I_{\lim}-I\right)}^{n},$$ (2)

where [P]_0_ is the initial concentration of the protein, *I*_0_ is the initial value of *I* and *I*_lim_ is the limiting value of *I* at *t* → ∞. The value of (*I*_lim_—*I*_0_) is proportional to the initial protein concentration: (*I*_lim_−*I*_0_) = ε[P]_0_.

When *n* = 1 (first order of aggregation), Eq 2 is transformed into linear anamorphosis:

$$\frac{d\left(I-I_{0}\right)}{dt}=k_{I}\left(I_{\lim}-I\right),$$ (3)

where *k*_I_ is the rate constant of the first order. Integration of Eq 3 yields an expression describing the dependence of *I* on time [1,16–19]:

$$I=I_{0}+(I_{\lim}-I_{0})\{1-\exp[-k_{I}(t-t_{0})]\},$$ (4)

where *t*_0_ is a length on the horizontal line *I* = *I*_0_ cut off by a theoretical curve calculated with this equation. The slope of a tangent to the theoretical curve passing through the point with coordinates {*t* = *t*_0_; *I* = *I*_0_} is equal to the product *k*_I_(*I*_lim_−*I*_0_). This product is a measure of the initial rate of aggregation.

If the kinetic curves are obtained at various initial protein concentrations, the following generalized linear anamorphosis can be constructed:

$$\frac{1}{{\left[P\right]}_{0}}\left(\frac{d(I-I_{0})}{dt}\right)=\varepsilon k_{I}-k_{I}\frac{\left(I-I_{0}\right)}{{\left[P\right]}_{0}}$$ (5)

If the aggregation process follows kinetics of the reaction of the first order, the experimental points obtained at various [P]_0_ values should fall on the common straight line in the coordinates {[d(*I*–*I*_0_)/d*t*]/[P]_0_; (*I*–*I*_0_)/[P]_0_}.

The initial stage of aggregation of many proteins is association of non-native protein molecules with the formation of nuclei capable of further growth via the attachment of monomeric unfolded protein [20,21]. It is significant that Eqs 3–5 are used for the description of the kinetic curves after termination of the nucleation stage, i.e., for the description of the growth stage of protein aggregates.

## Results

### UV-induced denaturation of Ph*b*

**Fig 1:**
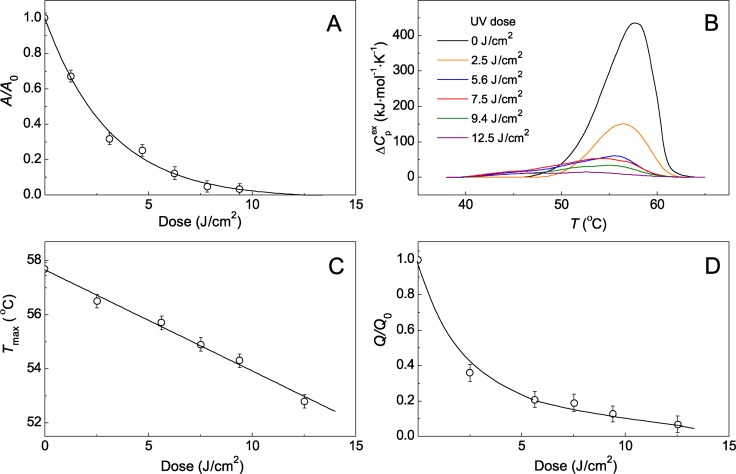
Enzymatic activity and heat stability of UV-Ph*b*. (A) Dependence of the relative enzymatic activity *A*/*A*_0_. (B) Dependences of the excess heat capacity ($\Delta C_{p}^{\mathrm{ex}}$) on temperature for intact Ph*b* and Ph*b* irradiated by different UV doses. The initial concentration of Ph*b* was 1.5 mg/ml. $\Delta C_{p}^{\mathrm{ex}}$ was calculated per Ph*b* dimer (*M*_r_ = 194800 Da). (C) Position of the maximum *T*_max_ on the DSC profiles. (D) Relative denaturation heat *Q*/*Q*_0_. Conditions of the experiments: 0.03 M Hepes buffer, pH 6.8, containing 0.1 M NaCl. *A*_0_ and *A* are the values of enzymatic activity for intact Ph*b* and UV-Ph*b*, respectively. *Q*_0_ and *Q* are the values of the area under DSC profile for intact Ph*b* and UV-Ph*b*, respectively. Three independent measurements were used to determine the arrow bars shown in this figure.

UV irradiation leads to a loss in enzymatic activity and destruction of the native structure of Ph*b*. Fig 1A shows a dependence of the relative enzymatic activity on radiation dose. As it can be seen from this figure, the enzymatic activity is practically undetected at the radiation dose of 9.4 J/cm^2^. Denaturation of Ph*b* during UV irradiation is supported by the data of DSC. DSC gives valuable information on protein stability [22,23]. Application of this method to study of UV-Ph*b* stability (Fig 1B) shows that there are the following changes in DSC profiles during UV irradiation. An increase in radiation dose is accompanied by a shift of the maximum position (*T*_max_) towards lower temperatures (Fig 1C) and the diminishing of the area under DSC profile which corresponds to denaturation heat (*Q*) and is proportional to the fraction of the native protein. In Fig 1D *Q*/*Q*_0_ ratio is represented as a function of radiation dose (*Q*_0_ is the value of *Q* for the intact protein). *Q*/*Q*_0_ value decreases to approximately 0.1 at radiation dose of 9.4 J/cm^2^.

To characterize the effect of UV irradiation on the oligomeric state of Ph*b*, sedimentation velocity was used. Increasing the UV irradiation time (dose) is accompanied by the disappearance of native Ph*b* dimeric form and appearance of the larger particles with higher sedimentation coefficients (S1 Fig and S1 Table). Comparison of sedimentation coefficient distributions (S1 Fig) and the average values of sedimentation coefficient represented in S1 Table shows that UV-Phb oligomeric state becomes more polydisperse and demonstrates the presence of higher-order oligomers with increasing time of UV-irradiation.

### Kinetics of UV-Ph*b* aggregation

**Fig 2:**
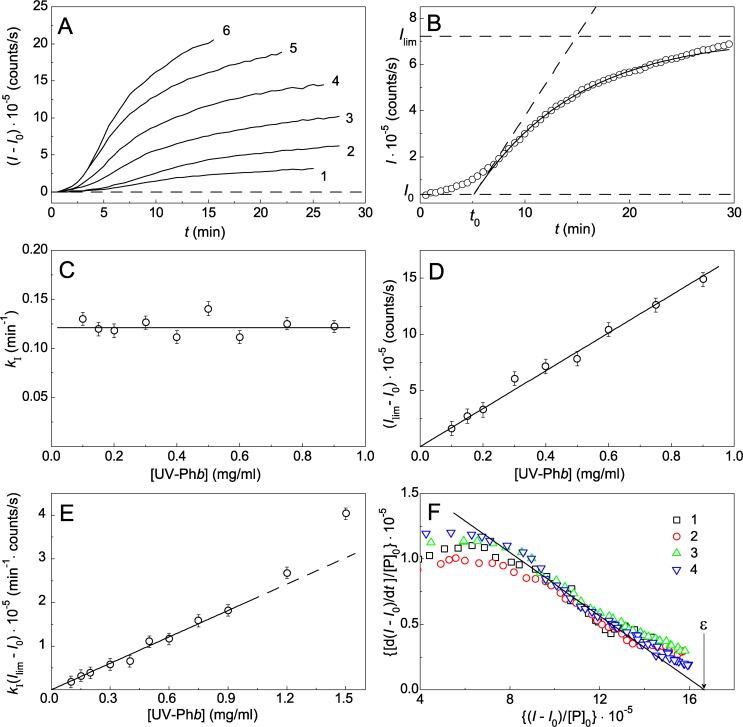
The kinetics of aggregation of UV-Ph*b* followed by the increase in the light scattering intensity at 37°C (radiation dose was 9.4 J/cm^2^). (A) The dependences of the light scattering intensity (*I–I*_0_) on time obtained at the following concentrations of UV-Ph*b*: 0.2 (1), 0.4 (2), 0.6 (3), 0.9 (4) 1.2 (5) and 1.5 mg/ml (6). *I* and *I*_0_ are the current and initial values of the light scattering intensity, respectively. (B) Fitting Eq 4 to the experimental data obtained at [UV-Ph*b*] = 0.4 mg/ml. Points are the experimental data. The solid curve was calculated at *k*_I_ = 0.115 min^-1^ and *t*_0_ = 5.54 min. The horizontal dashes correspond to *I*_0_ and *I*_lim_ values. (C, D and E) The dependences of *k*_I_, (*I*_lim_−*I*_0_) and *k*_I_(*I*_lim_−*I*_0_) values on the concentration of UV-Ph*b*. (F) The kinetic curves represented in coordinates {[d(*I—I*_0_)/d*t*]/[P]_0_; (*I—I*_0_)/[P]_0_}, where [P]_0_ is the concentration of UV-Ph*b*. The dimension of [d(*I—I*_0_)/d*t*]/[P]_0_ is [min^-1^·(counts/s)]/(mg/ml); the dimension of (*I—I*_0_)/[P]_0_ is (counts/s)/(mg/ml). The concentrations of UV-Phb were the following: 0.2 (1), 0.4 (2), 0.6 (3), 0.9 mg/ml (4). The solid line was calculated from Eq 5 at *k*_I_ = 0.122 min^-1^ and ε = 1.67·10^6^ (counts/s)/(mg/ml). Three independent measurements were used to determine the error bars shown in panels C, D and E.

DLS was used to study the kinetics of aggregation of Ph*b* irradiated with the dose of 9.4 J/cm^2^. The aggregation process was carried out at 37°C at different concentrations of the protein. At this temperature intact Ph*b* is relatively stable [24]. However, UV-Ph*b* reveals high propensity to aggregation at 37°C [2,25]. Fig 2A shows the dependences of the light scattering intensity (*I*) on time obtained for aggregation of UV-Ph*b* at 37°C. UV-Ph*b* concentration was varied in the interval from 0.2 to 1.5 mg/ml. The applicability of Eq 4 for description of the aggregation kinetics is demonstrated in Fig 2B for the kinetic curve obtained at [UV-Ph*b*] = 0.4 mg/ml. When fitting Eq 4 to the experimental data, the following values of parameters were obtained: *I*_0_ = 0.37·10^−5^ counts/s, *I*_lim_ = (7.23 ± 0.08)·10^5^ counts/s, *k*_I_ = 0.115 ± 0.004 min^-1^ and *t*_0_ = 5.54 ± 0.11 min (*R*^2^ = 0.9987). The slope of a tangent to the theoretical curve passing through the point with coordinates {*t* = *t*_0_; *I* = *I*_0_} is equal to the product *k*_I_(*I*_lim_−*I*_0_) = (0.785 ± 0.007)·10^5^ [min^-1^·(counts/s)] and characterizes the initial rate of aggregation. It should be noted that deviations from the calculated curve are observed at high values of time (*t* > 25 min). These deviations are probably due to sticking of the large-sized aggregates.

**Fig 3:**
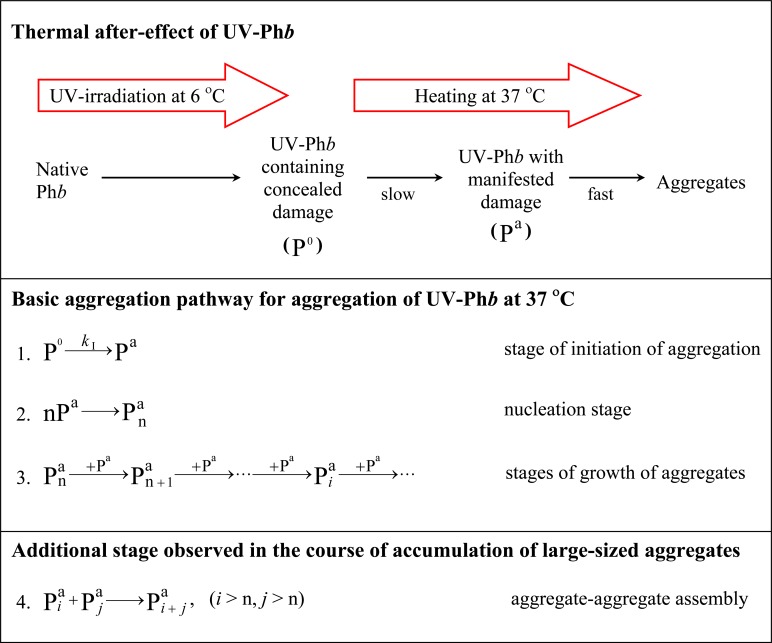
Scheme illustrating thermal after-effect of UV irradiation of Ph*b*. Heating of UV-Ph*b* with concealed damage (P^0^) at 37°C results in a structural reorganization of UV-irradiated protein to a state with manifested damage (P^a^). Slow transformation of P^0^ into P^a^ is followed by the nucleation stage and fast stage of aggregate growth with formation of amorphous aggregates. The growth of aggregates proceeds by attachment of P^a^ to the existing aggregates (basic aggregation pathway). Sticking of protein aggregates can be observed in the course of accumulation of large-sized aggregates (additional aggregation stage).

The analysis of the kinetic curves obtained at different concentrations of UV-Ph*b* allowed us to construct the dependences of parameters *k*_I_, (*I*_lim_—*I*_0_) and their product *k*_I_(*I*_lim_—*I*_0_) on the protein concentration (Fig 2C–2E, respectively). The parameter *k*_I_ remains practically constant at varying protein concentration (in the interval of the protein concentration from 0.1 to 0.9 mg/ml; Fig 2C). The average value of the reaction rate constant of the first order *k*_I_ is equal to 0.122 ± 0.008 min^-1^. Parameter (*I*_lim_−*I*_0_) is a linear function of UV-Ph*b* concentration (Fig 2D). The value of ε corresponding to the slope of the (*I*_lim_−*I*_0_) versus [UV-Ph*b*] plot was found to be (1.67 ± 0.07)·10^6^·(counts/s)/(mg/ml). The linear character of the dependence of (*I*_lim_−*I*_0_) on [UV-Ph*b*] supports the suggestion that the value of the light scattering intensity is proportional to the amount of the aggregated protein. As it can be seen from Fig 2E, the product *k*_I_(*I*_lim_−*I*_0_) which characterizes the initial rate of aggregation is proportional to UV-Ph*b* concentration. This means that the order of aggregation with respect to protein is equal to unity. Thus, constancy of *k*_I_ value at varying UV-Ph*b* concentration, and linear character of the initial rate of aggregation on the protein concentration testifies to the fact that aggregation of UV-Ph*b* at 37°C follows kinetics of the reaction of the first order. As for parameter *t*_0_, the *t*_0_ value decreases from 6.60 ± 0.30 to 2.53 ± 0.05 min, when the protein concentration rises from 0.1 to 1.5 mg/ml. Such aggregation kinetics means that in the UV-Ph*b* molecule the aggregation stage was preceded by the stage of heat-induced intramolecular structural transition. Slow structural transition in UV-damaged Ph*b* molecule is followed by the nucleation stage and fast stage of aggregate growth (Fig 3). Thus, parameter *k*_I_ can be interpreted as a reaction rate constant of the first order characterizing thermal after-effect of UV irradiation of Ph*b*.

Fig 2F shows the experimental data represented in the coordinates {[d(*I*–*I*_0_)/d*t*]/[P]_0_; (*I*–*I*_0_)/[P]_0_} used for construction of generalized linear anamorphosis represented by Eq 5. As expected, the points corresponding to the regions of the kinetic curves after the inflection point fall on the common straight line. This fact supports the conclusion that heat-induced aggregation of UV-Ph*b* at 37°C is of the first order with respect to the protein.

It should be noted that at relatively high protein concentrations (higher than approximately 1 mg/ml) the linear relationship between the initial rate of aggregation and UV-Ph*b* concentration breaks down (Fig 2E).

**Fig 4:**
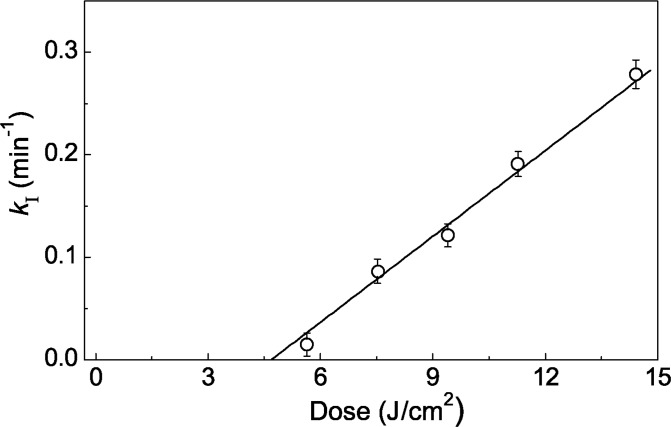
Propensity to aggregation of UV-Ph*b*. The reaction rate constant of the first order *k*_I_ calculated from Eq 4 for aggregation of UV-Ph*b* (0.3 mg/ml) on radiation dose. Three independent measurements were used to determine the arrow bars shown in this figure.

Fig 4 shows the dependence of *k*_I_ value on radiation dose. It is of interest that the rate of structural reorganization of UV-Ph*b* increases with radiation dose. When radiation dose increases from 5.6 to 14.4 J/cm^2^ a 18-fold increase in *k*_I_ value is observed.

### Relationship between the increment of the light scattering intensity and the amount of the aggregated protein

**Fig 5:**
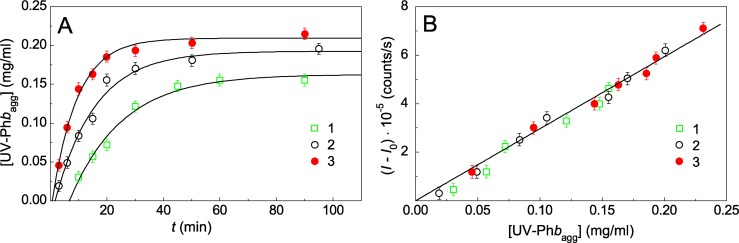
The comparison of the kinetics of aggregation of UV-Ph*b* (0.26 mg/ml) at 37°C followed by an increase in the concentration of aggregated UV-Ph*b* and increase in the light scattering intensity. (A) The time dependences of the concentration of aggregated UV-Ph*b* irradiated with the following doses: 7.5 (1), 9.4 (2) and 12.5 J/cm^2^ (3). The points are the experimental data. The solid curve was calculated from Eq 6 at the following values of parameters: *t*_0_ = 6 min, *k*_I_ = 0.074 min^-1^ and [UV-Ph*b*_agg_]_lim_ = 0.16 mg/ml for curve 1; *t*_0_ = 1.71 min, *k*_I_ = 0.107 min^-1^ and [UV-Ph*b*_agg_]_lim_ = 0.19 mg/ml for curve 2; *t*_0_ = 0.67 min, *k*_I_ = 0.139 min^-1^ and [UV-Ph*b*_agg_]_lim_ = 0.21 mg/ml for curve 3. (B) The relationship between increment of the light scattering intensity (*I*–*I*_0_) and the concentration of aggregated UV-Ph*b*. The radiation doses were the following: 7.5 (1), 9.4 (2) and 12.5 J/cm^2^ (3). The concentration of aggregated UV-Ph*b* was determined from measurements of optical density of supernatant at 280 nm after precipitation of protein aggregates by centrifugation (see Methods). The error bars were calculated using three independent measurements.

Eq 4 was derived on the assumption that the light scattering intensity registered in the course of protein aggregation is proportional to the concentration of the aggregated protein. To check for existence of such a proportionality, we compared an increment in the light scattering intensity with the concentration of aggregated UV-Ph*b* (UV-Ph*b*_agg_) which was calculated from experiments involving centrifugation of UV-Ph*b* solutions preheated for different time intervals at 37°C. Fig 5A shows the dependences of [UV-Ph*b*_agg_] on time for aggregation of UV-Ph*b* preparations (0.26 mg/ml) irradiated with dose of 7.5, 9.4 and 12.5 J/cm^2^. The following equation was used for description of these kinetic curves:

$$[\mathrm{UV}\text{-}\mathrm{Ph}b_{\mathrm{agg}}]={\left[\mathrm{UV}\text{-}\mathrm{Ph}b_{\mathrm{agg}}\right]}_{\lim}\{1-\exp[-k_{I}(t-t_{0})]\},$$ (6)

where [UV-Ph*b*_agg_]_lim_ is the limiting value of [UV-Ph*b*_agg_] at *t* → ∞. As it can be seen from the figure, the experimental data are satisfactorily described by Eq 6. The following values for the reaction rate constant *k*_I_ were obtained: *k*_I_ = 0.074 ± 0.008, 0.107 ± 0.009 and 0.139 ± 0.014 min^-1^ at radiation dose of 7.5, 9.4 and 12.5 J/cm^2^, respectively. It is significant that the obtained values of the reaction rate constant *k*_I_ coincide with the corresponding values determined from the light scattering intensity measurements. The construction of the light scattering intensity versus [UV-Ph*b*_agg_] plot (Fig 5B) demonstrates the existence of proportionality between the increment of the light scattering intensity and concentration of the aggregated protein. It should be noted that such proportionality was also established for heat-induced aggregation of intact Ph*b* at 48°C [26].

In the experiments represented in Fig 5A the concentration of UV-Ph*b* [UV-Ph*b*]_0_ was 0.26 mg/ml. It should be noted that the values of [UV-Ph*b*_agg_]_lim_ are less than [UV-Ph*b*]_0_: [UV-Ph*b*_agg_]_lim_/[UV-Ph*b*]_0_ = 0.62, 0.74 and 0.80 for Ph*b* preparations irradiated with dose of 7.5, 9.4 and 12.5 J/cm^2^, respectively. This means that a part of UV-Ph*b* remains in the non-aggregated state over 90 min of heating at 37°C.

**Fig 6:**
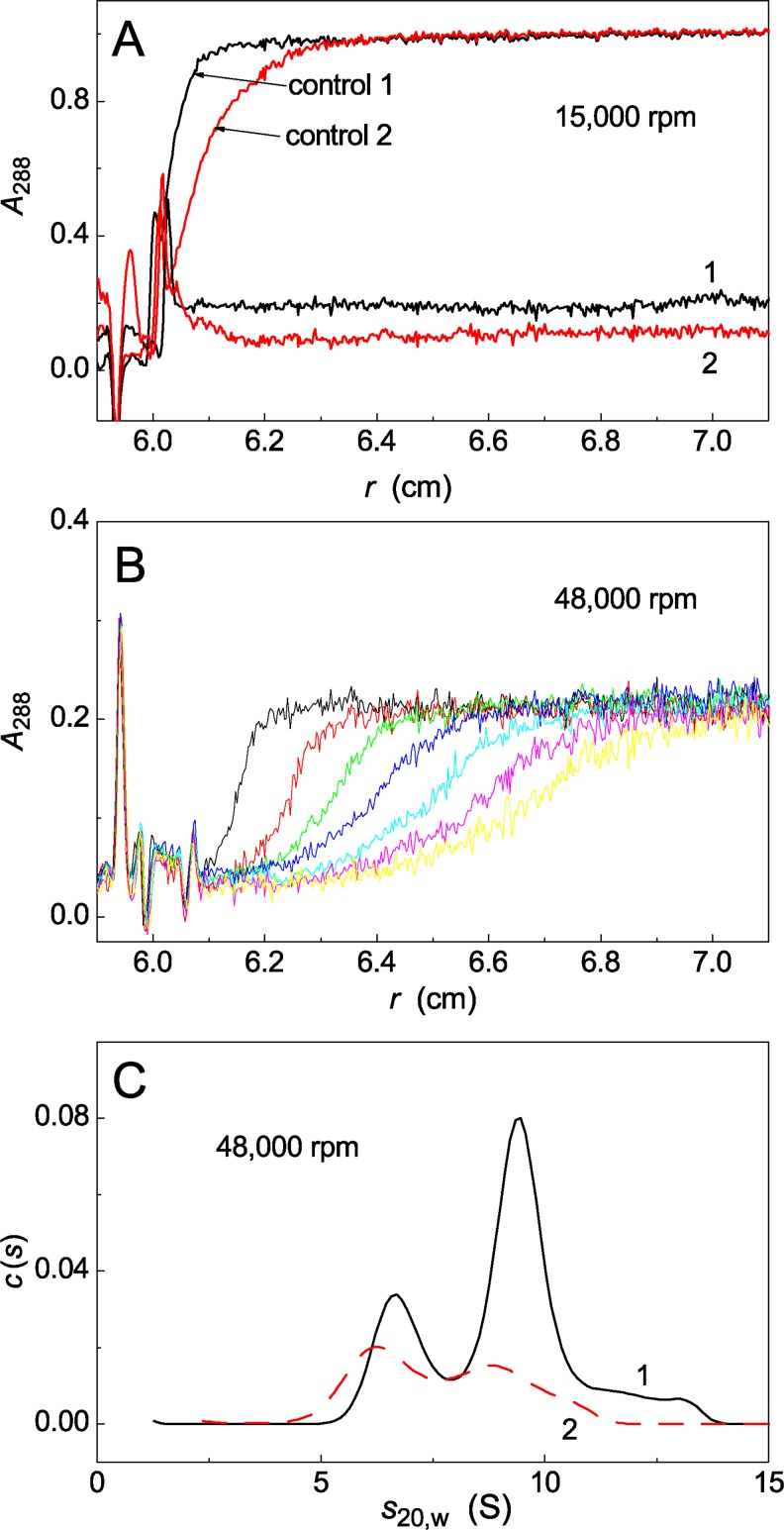
Sedimentation behaviour of UV-Ph*b* (0.75 mg/ml) at 25°C. The samples were heated at 37°C for 90 min and cooled to 25°C after heating. (A) Sedimentation profiles for Ph*b* irradiated with dose of 9.4 (sample 1) and 12.5 J/cm^2^ (sample 2) before heating. *A*_288_ is absorbance at 288 nm, *r* is radial distance. Control curves (control 1 and control 2) correspond to non-heated UV-Ph*b* (radiation doses were 9.4 and 12.5 J/cm^2^, respectively). Sedimentation runs were carried out at 25°C. Rotor speed was 15,000 rpm. (B) Sedimentation profiles for sample 1 (Ph*b* irradiated with dose of 9.4 J/cm^2^ before heating) at 25°C. Rotor speed was 48,000 rpm. Every 4th scan was taken for presentation. (C) The *c*(*s*) sedimentation coefficient distributions obtained at 25°C were transformed to standard *s*_20,w_ distributions for samples 1 and 2. Sedimentation runs were carried out at 25°C. Rotor speed was 48,000 rpm.

It was of interest to analyze the preheated UV-Ph*b* preparations by sedimentation velocity method. The sedimentation data represented in Fig 6A confirm our conclusion that UV-Ph*b* preparations heated for 90 min at 37°C contain the non-aggregated forms of the protein. The rotor speed was set at 15,000 rpm. Comparison of the optical absorbances of sedimentation profiles in the plateau region for unheated UV-Ph*b* (0.75 mg/ml; control 1 and control 2) and preheated UV-Ph*b* (0.75 mg/ml; samples 1 and 2) shows that full precipitation of large-sized aggregates takes place under given conditions (designations 1 and 2 correspond to radiation dose of 9.4 and 12.5 J/cm^2^, respectively). These data allow us to calculate the fraction of the protein remaining in the non-aggregated state over 90 min of heating: 0.19 and 0.11 for Ph*b* irradiated with dose of 9.4 and 12.5 J/cm^2^, respectively. To characterize the fraction of the non-aggregated protein, sedimentation velocity runs were performed at higher rotor speed (48,000 rpm; Fig 6B and 6C). The *c*(*s*) distribution shows that in sample 1 the non-aggregated protein exists as small-sized oligomers with sedimentation coefficients *s*_20,w_ of 6.6, 9.4 and 13 S (Fig 6C, curve 1) which might correspond to the monomeric, dimeric and tetrameric forms, respectively. There are peaks with *s*_20,w_ of 6.2 and 8.9 S on the *c*(*s*) distribution for sample 2 (Fig 6C, curve 2).

### Determination of the activation energy for the after-effect of UV irradiation of Ph*b*

**Fig 7:**
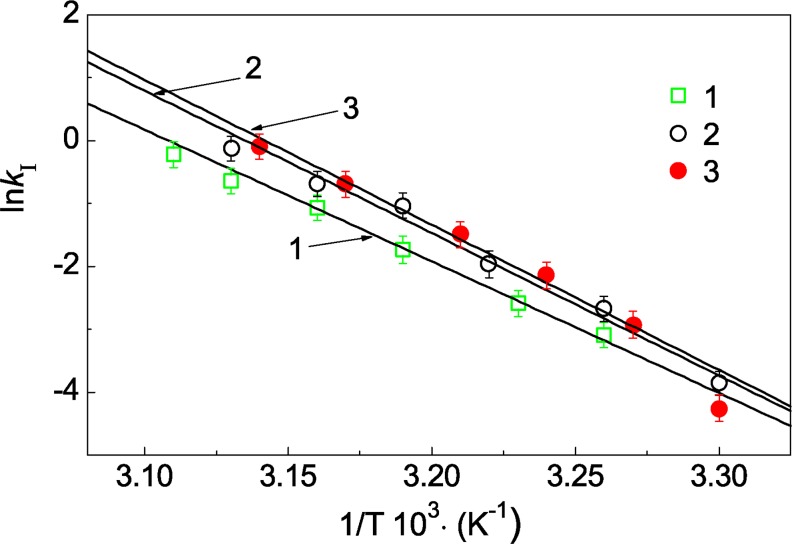
The determination of the activation energy for aggregation of UV-Ph*b* (0.3 mg/ml). The dependences of ln*k*_I_ on the reciprocal value of the absolute temperature for aggregation of UV-Ph*b* irradiated with the following doses: 7.5 (1), 9.4 (2) and 12.5 J/cm^2^ (3). The reaction rate constant *k*_I_ was calculated using Eq 4. The error bars were calculated using three independent measurements.

The measurements of the reaction rate constant *k*_I_ at different temperatures allowed us to determine the activation energy for heat-induced structural rearrangement of UV-Ph*b*. Fig 7 shows the dependence of ln*k*_I_ on the reciprocal value of the absolute temperature for Ph*b* preparations irradiated with dose of 7.5, 9.4 and 12.5 J/cm^2^. The linear character of these dependences indicates that the Arrhenius equation can be used to describe the influence of temperature on the after-effect of UV irradiation of Ph*b* [27]:

$$k_{I}=\exp\left[\frac{E_{a}}{R}\left(\frac{1}{T^{*}}-\frac{1}{T}\right)\right]\min^{-1},$$ (7)

where *E*_a_ is the experimental activation energy, *R* is a gas constant (*R* = 0.00831 kJ/grad·mol and T* is the absolute temperature at which *k*_I_ = 1 min^-1^. The following values for parameters of the Arrhenius equation are found: *E*_a_ = 164 ± 9 kJ/mole and T* = 322.6 ± 0.2 K (*R*^2^ = 0.9872) for radiation dose of 7.5 J/cm^2^, *E*_a_ = 188 ± 11 kJ/mole and T* = 318.5 ± 0.2 K (*R*^2^ = 0.9871) for radiation dose of 9.4 J/cm^2^, *E*_a_ = 215 ± 12 kJ/mole and T* = 317.5 ± 0.2 K (*R*^2^ = 0.9854) for radiation dose of 12.5 J/cm^2^.

### Thermal stability of UV-Ph*b* characterized by CD

**Fig 8:**
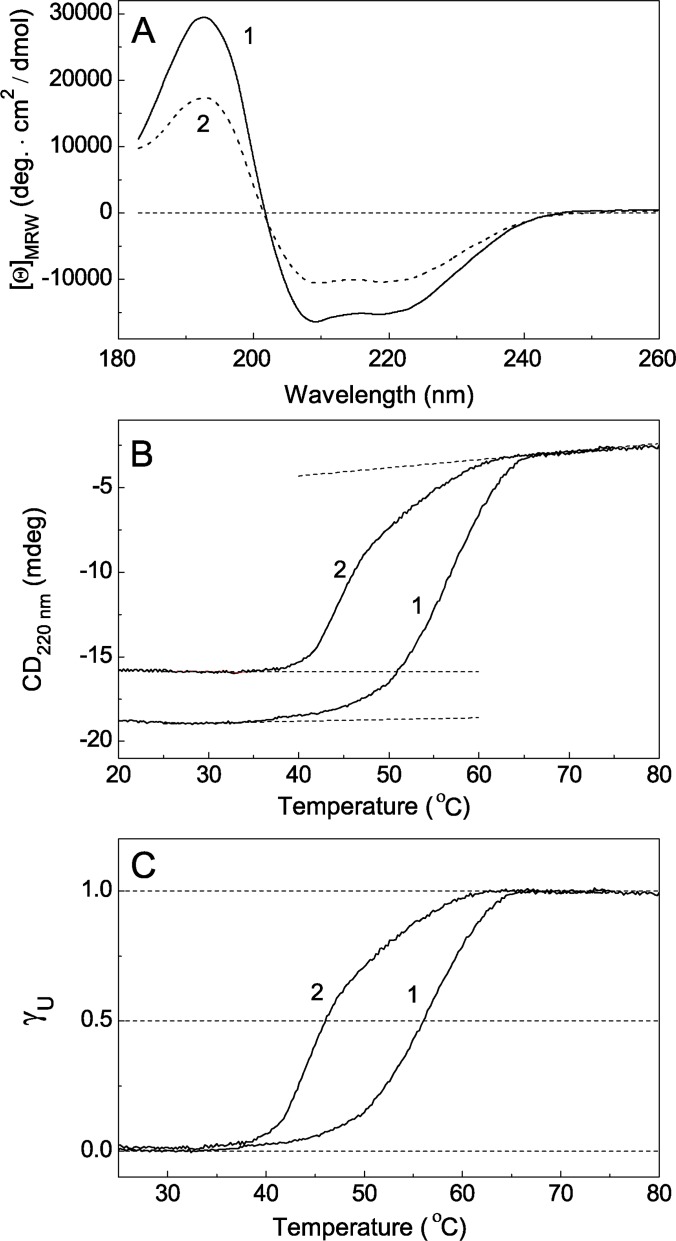
A comparison of the properties of intact Ph*b* and UV-Ph*b* using CD. (A) The CD spectra for intact Ph*b* (1 mg/ml, curve 1) and Ph*b* irradiated with the dose of 9.4 J/cm^2^ (1 mg/ml, curve 2) at 10°C. (B) The dependence of ellipticity at 220 nm and (C) the portion of unfolded protein (γ_U_) on temperature for intact Ph*b* (0.17 mg/ml, curve 1) and UV-Ph*b* (0.17 mg/ml, curve 2). The values of γ_U_ were calculated using Eq 8. The dashed horizontal lines on panel B correspond to the asymptotes for the regions of relatively low and relatively high temperatures. The dashed horizontal lines on panel C correspond to the γ_U_ values equal to 0, 0.5 and 1.0.

Fig 8A shows CD spectra of intact Ph*b* (curve 1; 1 mg/ml) and UV-Ph*b* (curve 2; 1 mg/ml) at 10°C. A calculation of the content of major elements of the secondary structure shows that the observed change in the spectra is due to a 1.65-fold decrease in the portion of α-helixes (from 0.53 to 0.32), 2.3-fold increase in the portion of β-strands (from 0.09 to 0.21), 1.1-fold increase in turns (from 0.12 to 0.13) and 1.2-fold increase in unordered structure elements (from 0.27 to 0.33) (Table 1).

**Table 1:** Change in the content of the main elements of the secondary structure of glycogen phosphorylase *b* after UV irradiation.

Sample	α-helixes	β-strands	Turns	Unordered	Total
**Ph*b***	0.53	0.09	0.12	0.27	1.01
**UV-Ph*b***	0.32	0.21	0.13	0.33	0.99

Heat-induced structural transitions in intact Ph*b* (0.17 mg/ml) and Ph*b* irradiated with the dose of 9.4 J/cm^2^ (0.17 mg/ml) were registered by monitoring the elipticity change at 220 nm (Fig 8B). Unfolding of the protein molecules is accompanied by the increase in elipticity at 220 nm. To describe the dependence of elipticity at 220 nm (*y*) on temperature (*T*) the following equation can be used [28]:

$$y=y_{N}+m_{N}T+(y_{U}+m_{U}T-y_{N}-m_{N}T)\gamma_{U},$$ (8)

In this equation γ_U_ are the molar fraction of the native and unfolded states, respectively. The pre- and posttranslational baselines were defined by (*y*_N_ + *m*_N_*T*) and (*y*_U_ + *m*_U_*T*), where *y*_N_, *m*_N_, *y*_U_ and *m*_U_ are constants. An analogous equation can be applied for the description of temperature-induced unfolding of UV-Ph*b*. The dependences of molar fraction of unfolded protein (γ_U_) on temperature for intact Ph*b* and UV-Ph*b* (Fig 8C, curves 1 and 2, respectively) are obtained with this equation. Thermal stability of a protein can be characterized by parameter *T*_0.5_, i.e. the temperature at which γ_U_ = 0.5. The following values for parameter *T*_0.5_ were found for intact Ph*b* and Ph*b* irradiated with the dose of 9.4 J/cm^2^: *T*_0.5_ = 56.6 and 46.2°C, respectively. These data demonstrate that UV-Ph*b* reveals remarkably lower thermal stability than intact Ph*b*.

It is of interest that the dependence of γ_U_ on temperature for UV-Ph*b* (curve 2 in Fig 8C) involves two temperature-induced transitions. One may assume that the first transition (at temperatures lower than ~47°C) corresponds to the structural reorganization of UV-Ph*b* containing concealed damage, whereas the second transition (at temperatures higher than ~47°C) is related to the unfolding of a protein fraction which remained in the non-aggregated state in the aggregation experiments at 37°C (Figs 3 and 5). It should be noted that γ_U_ value is really the sum of two terms corresponding to two unfolded states.

### Analysis of the hydrodynamic radii (*R*_h_) of protein aggregates formed in the course of UV-Ph*b* aggregation

**Fig 9:**
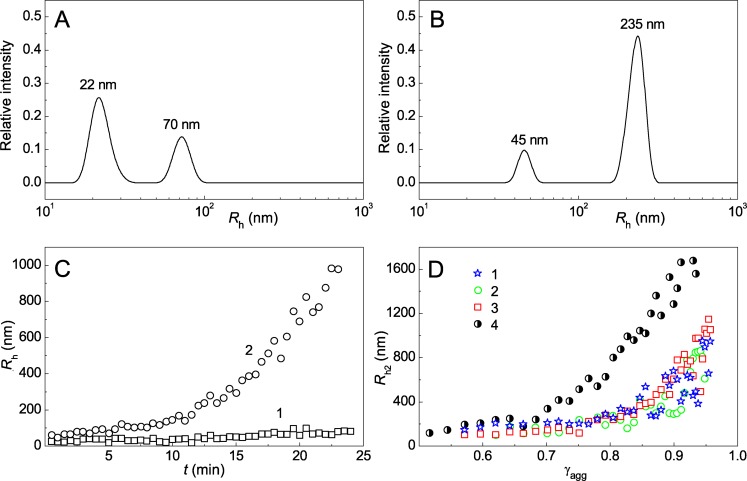
The changes in the size of the protein aggregates formed in the process of UV-Ph*b* aggregation at 37°C. (A and B) The distribution of particles by their size registered for UV-Ph*b* (0.4 mg/ml) heated for 0.5 and 13.5 min, respectively. (C) The dependences of the hydrodynamic radius (*R*_h_) on time for small-sized (1) and large-sized aggregates (2) ([UV-Ph*b*] = 0.5 mg/ml). (D) The *R*_h,2_ value versus γ_agg_ plots obtained at the following concentrations of UV-Ph*b*: 0.15 (1), 0.3 (2), 0.5 (3) and 1.2 mg/ml (4). *R*_h,2_ is the hydrodynamic radius of large-sized aggregates. γ_agg_ is the fraction of the aggregated protein calculated from Eq 9 (0.03 M Hepes buffer, pH 6.8, containing 0.1 M NaCl; radiation dose was 9.4 J/cm^2^).

DLS used for registration of UV-Ph*b* aggregation at 37°C allows determining the size of protein particles formed in the aggregation process. The size particle distribution for heated UV-Ph*b* manifests bimodal character at the early stages of aggregation. As it can be seen from Fig 9A, there are two peaks in the particle size distribution (*R*_h,1_ = 22 nm and *R*_h,2_ = 70 nm) for UV-Ph*b* (0.5 mg/ml) heated for 0.5 min at 37°C (radiation dose was 9.4 J/cm^2^). The increase in the size of both peaks is observed in the course of heating. There are peaks with *R*_h,1_ = 45 nm and *R*_h,2_ = 235 nm in the particle size distribution for UV-Ph*b* heated for 13.5 min (Fig 9B). Fig 9C shows the dependence of *R*_h,1_ and *R*_h,2_ on time. The changes in the *R*_h,2_ value are markedly higher than those for *R*_h,1_ value. It is evident that heavier particles with *R*_h_ = *R*_h,2_ make the greatest contribution to the light scattering intensity of the protein solution.

Valuable information on the mechanism of protein aggregation can be obtained from the dependences of the hydrodynamic radius on the fraction of the aggregated protein (γ_agg_). The *R*_h_ versus γ_agg_ plot was constructed for the *R*_h_ values characterizing the heavier component of UV-Ph*b* aggregates (*R*_h,2_). Taking into account the first order of UV-Ph*b* aggregation with respect to the protein, we calculated the fraction of aggregated protein using the following equation:

$$\gamma_{\mathrm{agg}}=1-\exp[-k_{I}(t-t_{0})].$$ (9)

As it can be seen from Fig 9D, the experimental points corresponding to different protein concentrations (0.1–0.5 mg/ml) fall on the common curve. These data coincide with the aggregation mechanism represented in Fig 3. The initial stage of the overall process of aggregation (stage 1) is the formation of UV-Ph*b* with manifested damage (P^a^). This protein form acts as a main building block in the aggregation process. After the completion of the nucleation stage the growth of aggregates proceeds by attachment of P^a^ form to the existing aggregates (stage 3). The placement of the experimental points in the coordinates {*R*_h_; γ_agg_} on the common curve means that there is no sticking of the formed aggregates. It should be noted that at relatively high protein concentrations deviations from the basic aggregation pathway can be observed due to aggregate–aggregate assembly (stage 4). For example, such deviations are observed for *R*_h,2_ versus γ_agg_ plot constructed at [UV-Ph*b*] = 1.2 mg/ml (curve 4 in Fig 9D).

## Discussion

UV irradiation of proteins causes damage to the secondary structure, exposure of hydrophobic residues, unfolding and aggregation [29–31]. It is generally accepted that UV-irradiated denaturation of proteins follows a one-hit model. According to this model denaturation of a protein molecule proceeds in line with the all-or-none principle as a result of the absorption of one resulting photon [29,32–33]. The validity of the one-hit model can be confirmed by DSC data. When the position of *T*_max_ remains unchanged at varying radiation dose, the one-hit model is applicable. The latter is observed, for example, in the case of UV-irradiated β_L_-crystallin [34] and UV-irradiated α-crystallin [35]. However, UV irradiation of Ph*b* is accompanied by a shift of *T*_max_ towards lower temperatures with increasing radiation dose (Fig 1C). Thus, UV induces formation of damaged forms of Ph*b* possessing lesser thermal stability. According to DSC data, damaged protein states, which are less thermally stable, develop in the course of UV irradiation of GAPDH [3]. J.H. Clark [36] was the first to demonstrate a decrease in thermal stability of a protein (egg albumin) irradiated by ultra violet. Subsequently, it was shown that thermal inactivation of UV-irradiated lactate dehydrogenase [37] and GAPHD [3] from rabbit skeletal muscles and trypsin [38] proceeds with higher rate than that of the intact enzymes.

Analysis of kinetics of UV-Ph*b* aggregation at 37°C allowed us to establish the kinetic regime of the aggregation process. Slow structural reorganization of UV-Ph*b* containing concealed damage is followed by fast aggregation of UV-Ph*b* forms with manifested damage (Fig 3). The structural rearrangement of UV-Ph*b* proceeds as a monomolecular process characterized by the reaction rate constant of the first order *k*_I_. It is significant that *k*_I_ value increases with increasing the radiation dose (Fig 4). The values of the activation energy (*E*_a_) for structural rearrangement of UV-Ph*b* measured from the temperature dependence of the reaction rate constant *k*_I_ are typical of those calculated for proteins undergoing one-step thermal denaturation (170–850 kJ/mol) [39–41].

When discussing the thermal after-effect of UV irradiation of Ph*b* it is essential to note that this enzyme belongs to a class of oligomeric proteins, the unfolding of which is under the control of a conformational lock between neighboring subunits [42,43]. The conformational lock provides for a multi-step process of breakdown of the oligomeric structure at elevated temperatures. One may assume that the conformational lock in a UV-Ph*b* molecule contains damage which causes its decreased stability upon heating.

The order of aggregation with respect to protein for aggregation of UV-Ph*b* at 37°C is equal to unity. This means that the rate-limiting stage of the overall aggregation process is the stage of UV-Ph*b* structural reorganization. The construction of *R*_h,2_ versus γ_agg_ plot (Fig 9D) allows us to conclude that the basic aggregation pathway for UV-Ph*b* is realized by the attachment of a main building block (P^a^) to the existing aggregates (Fig 3).

## Conclusion

A test system based on aggregation of UV-irradiated Ph*b* has been used to estimate the anti-aggregation activity of protein and chemical chaperones [2,25,44]. It is significant that this test system allows estimating anti-aggregation activity of the agents under study at physiological temperatures. In the present work a kinetic regime of UV-Ph*b* aggregation has been established, and it was demonstrated that the aggregation process involves a slow monomolecular stage of structural reorganization of UV-Ph*b* molecule followed by the nucleation stage and a fast stage of aggregate growth. It is evident that the analysis of the protective action of different agents using a test system based on UV-Ph*b* aggregation should be carried out with regard to the kinetic regime of the aggregation process. For example, if we study the protective action of agents which form reversibly dissociating complexes with UV-Ph*b*, the observed changes in the aggregation rate will be due to modulation of the rate-limiting stage, namely the stage of UV-Ph*b* structural transition, by the agents under study [45]. In conclusion, it should be noted that the fact of formation of protein states containing UV-induced concealed damage should be taken into account in all studies of the action of ultra violet on biological systems [46].

## Supporting information

S1 Checklist The ARRIVE guidelines checklist for reporting animal data in manuscript “A Thermal after-effect of UV irradiation of muscle glycogen phosphorylase *b*”. (PDF)

S1 Fig Sedimentation behavior of native Ph*b* and UV-Ph*b* (0.75 mg/ml). The *c*(*s*) distribution for native Ph*b* and ls-g*(*s*) sedimentation coefficient distributions for UV-irradiated Ph*b* were obtained at 20°C and transformed to standard *s*_20,w_ distributions. Inset is the same plot with expanded scale of ordinate axis. Rotor speed was 52000 rpm.
(PDF)

S1 Table The effect of UV irradiation on oligomeric state of Ph*b* (0.03 M Hepes, pH 6.8, 0.1 M NaCl, 20°C). (PDF)
