# Supplementary material for: A thermal after-effect of UV irradiation of muscle glycogen phosphorylase b
Source: PLoS One. 2017 Dec 7;12(12):e0189125. doi: 10.1371/journal.pone.0189125 (PMC5720721; doi:10.1371/journal.pone.0189125)
Supplement: S1 Fig — The c(s) distribution for native Phb and ls-g*(s) sedimentation coefficient distributions for UV-irradiated Phb were obtained at 20°C and transformed to standard s20,w distributions. Inset is the same plot with expanded scale of ordinate axis. Rotor speed was 52000 rpm. (PDF) [file pone.0189125.s002.pdf]

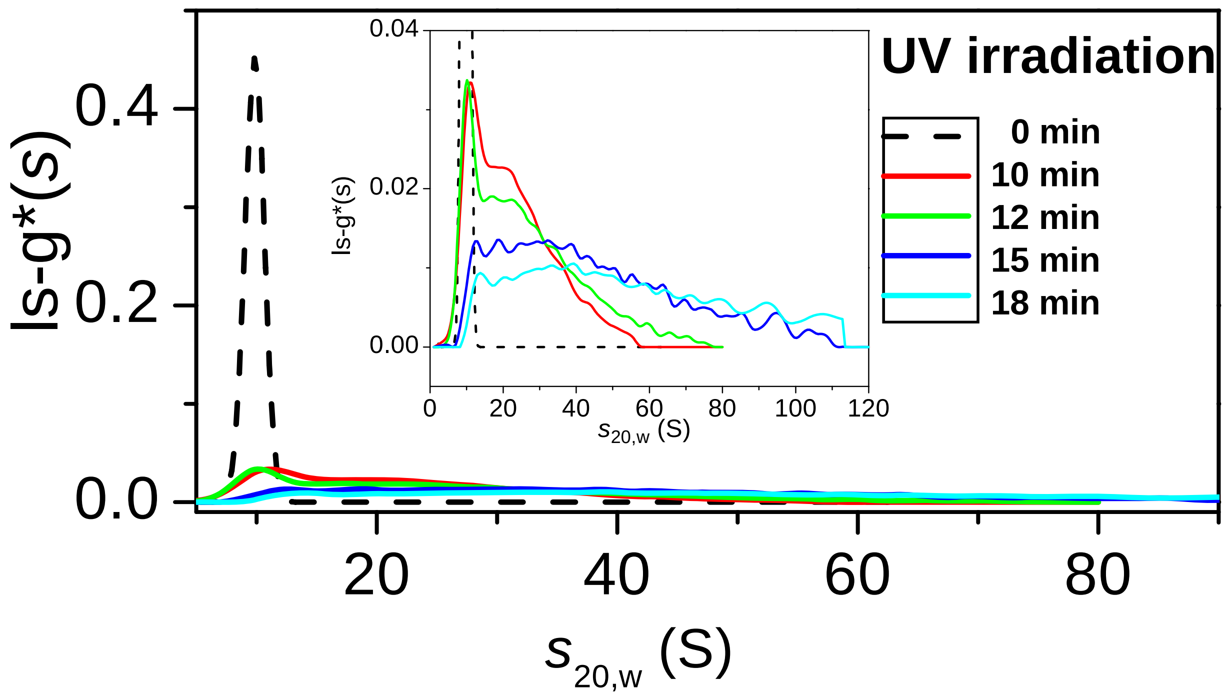

S1 Fig. Sedimentation behavior of native Ph*b* and UV-Ph*b* (0.75 mg/ml). The *c*(*s*) distribution for native Ph*b* and ls-g*(*s*) sedimentation coefficient distributions for UV-irradiated Ph*b* were obtained at 20 °C and transformed to standard $s_{20,w}$ distributions. Inset is the same plot with expanded scale of ordinate axis. Rotor speed was 52000 rpm.
