# Supplementary material for: A thermal after-effect of UV irradiation of muscle glycogen phosphorylase b
Source: PLoS One. 2017 Dec 7;12(12):e0189125. doi: 10.1371/journal.pone.0189125 (PMC5720721; doi:10.1371/journal.pone.0189125)
Supplement: S1 Table — (PDF) [file pone.0189125.s003.pdf]

S1 Table. The effect of UV irradiation on oligomeric state of Ph*b* (0.03 M Hepes, pH 6.8, 0.1 M NaCl, 20 °C)

	**Time of UV irradiation (min)**	**Dose of UV irradiation (J/cm^2)**	**s_{av} (S)**	**Std. Deviation (S)**	**R_h (nm)**
**Native Ph*b***	0	0	9.7	0.6	3.6
**UV-Ph*b***	10	6.3	24	11	5.6
**UV-Ph*b***	12	7.5	28	14	6.0
**UV-Ph*b***	15	9.4	47	24	7.9
**UV-Ph*b***	18	11.3	56	27	8.6

s_{av} is average value of sedimentation coefficient, Std. Deviation is standard deviations and R_h is calculated hydrodynamic radius.
